# North Atlantic subtropical mode water formation controlled by Gulf Stream fronts

**DOI:** 10.1093/nsr/nwad133

**Published:** 2023-05-08

**Authors:** Bolan Gan, Jingjie Yu, Lixin Wu, Gokhan Danabasoglu, R Justin Small, Allison H Baker, Fan Jia, Zhao Jing, Xiaohui Ma, Haiyuan Yang, Zhaohui Chen

**Affiliations:** Frontier Science Center for Deep Ocean Multispheres and Earth System (FDOMES) and Physical Oceanography Laboratory, Ocean University of China, Qingdao 266100, China; Laoshan Laboratory, Qingdao 266200, China; Frontier Science Center for Deep Ocean Multispheres and Earth System (FDOMES) and Physical Oceanography Laboratory, Ocean University of China, Qingdao 266100, China; Frontier Science Center for Deep Ocean Multispheres and Earth System (FDOMES) and Physical Oceanography Laboratory, Ocean University of China, Qingdao 266100, China; Laoshan Laboratory, Qingdao 266200, China; National Center for Atmospheric Research, Boulder, CO 80305, USA; National Center for Atmospheric Research, Boulder, CO 80305, USA; National Center for Atmospheric Research, Boulder, CO 80305, USA; Laoshan Laboratory, Qingdao 266200, China; CAS Key Laboratory of Ocean Circulation and Waves, Institute of Oceanology, Chinese Academy of Sciences, Qingdao 266071, China; Frontier Science Center for Deep Ocean Multispheres and Earth System (FDOMES) and Physical Oceanography Laboratory, Ocean University of China, Qingdao 266100, China; Laoshan Laboratory, Qingdao 266200, China; Frontier Science Center for Deep Ocean Multispheres and Earth System (FDOMES) and Physical Oceanography Laboratory, Ocean University of China, Qingdao 266100, China; Laoshan Laboratory, Qingdao 266200, China; Frontier Science Center for Deep Ocean Multispheres and Earth System (FDOMES) and Physical Oceanography Laboratory, Ocean University of China, Qingdao 266100, China; Laoshan Laboratory, Qingdao 266200, China; Frontier Science Center for Deep Ocean Multispheres and Earth System (FDOMES) and Physical Oceanography Laboratory, Ocean University of China, Qingdao 266100, China; Laoshan Laboratory, Qingdao 266200, China

**Keywords:** North Atlantic subtropical mode water, Gulf Stream thermal fronts, frontal-scale ocean-to-atmosphere feedback, eddy-resolving coupled global climate model

## Abstract

The North Atlantic Ocean hosts the largest volume of global subtropical mode waters (STMWs) in the world, which serve as heat, carbon and oxygen silos in the ocean interior. STMWs are formed in the Gulf Stream region where thermal fronts are pervasive and result in feedback with the atmosphere. However, their roles in STMW formation have been overlooked. Using eddy-resolving global climate simulations, we find that suppressing local frontal-scale ocean-to-atmosphere (FOA) feedback leads to STMW formation being reduced almost by half. This is because FOA feedback enlarges STMW outcropping, attributable to the mixed layer deepening associated with cumulative excessive latent heat loss due to higher wind speeds and greater air-sea humidity contrast driven by the Gulf Stream fronts. Such enhanced heat loss overshadows the stronger restratification induced by vertical eddies and turbulent heat transport, making STMW colder and heavier. With more realistic representation of FOA feedback, the eddy-present/rich coupled global climate models reproduce the observed STMWs much better than the eddy-free ones. Such improvement in STMW production cannot be achieved, even with the oceanic resolution solely refined but without coupling to the overlying atmosphere in oceanic general circulation models. Our findings highlight the need to resolve FOA feedback to ameliorate the common severe underestimation of STMW and associated heat and carbon uptakes in earth system models.

## Teaser

Frontal-scale ocean-to-atmosphere feedback determines the formation of subtropical mode water in the North Atlantic.

## INTRODUCTION

Subtropical mode waters (STMWs) are upper-ocean voluminous water masses characterized by vertically homogeneous temperature and salinity, and originate from the wintertime deep mixed layer on the warm flank of the western boundary current systems [1]. In the global ocean, the North Atlantic Ocean contains the thickest and volumetrically largest STMW, also known as the Eighteen Degree Water, hosting ∼43% of the total volume of global accumulation [2]. Capped by the surface stratification in spring, the mode water, with properties preserved, subducts into the main pycnocline and distributes widely in the western subtropical gyre [3]. The formation and ventilation of a large volume of STMW have far-reaching ramifications for climate and marine ecosystems. These include: storing and transporting heat [4,5]; injecting organic carbon and oxygen into the deep mesopelagic zone [6,7]; and acting as a complex nutrient supplier in the oligotrophic area [8,9]. Additionally, STMW constitutes an important sink for anthropogenic carbon dioxide into the ocean interior, acting as a buffer for climate change [10–12].

Given the importance of STMW with regard to memorizing climate variations and regulating ocean biogeochemical cycles, the physics governing its formation and destruction has received much attention. Traditionally, it has been considered that the bowl shape of STMW south of the Gulf Stream is constructed by wintertime intense convective mixing and eroded by diapycnal mixing [13–16]. The basin-scale atmospheric forcing linked to the North Atlantic Oscillation is typically regarded as controlling the renewal strength and carbon dioxide uptake capability of the mode water [11,17,18]. Due to the limitations resulting from sparse sampling of observations and coarse resolution of climate models, less knowledge has been acquired about the role of the Gulf Stream fronts and eddies in STMW production.

High-resolution ocean models provide some insight into the Ekman-driven convection induced by winds blowing in the downstream direction of oceanic thermal fronts [19,20], the mixed layer restratification by lateral heat fluxes and submesoscale processes [21,22] during the course of STMW formation, and the contribution of mesoscale eddies to subduction [23]. On the other hand, satellite observations and high-resolution ocean-atmosphere coupled model simulations indicate large amounts of heat release from sharp sea surface temperature (SST) fronts [24], leaving imprints on surface wind, storm track and vertical motion in the free atmosphere [25–27]. How ocean-to-atmosphere feedback at the scale of the Gulf Stream thermal fronts influences STMW production remains unknown. Here, we investigate this question using a state-of-the-art eddy-resolving coupled global climate model (CGCM), as coarse-resolution CGCMs have a poorly resolved Gulf Stream, severely underestimating the STMW formation rate [28].

## RESULTS

### Increased STMW production due to FOA feedback

To quantify the contribution of frontal-scale ocean-to-atmosphere (FOA) feedback to STMW production, a set of twin eddy-resolving global climate simulations were conducted using the Community Earth System Model (CESM [29]; see Model configuration and experimental design in Methods, Supplementary Data). One is a high-resolution fully coupled simulation [30] (referred to as CTRL), while the other is an identical simulation except that a spatial low-pass filter is applied to SST before being passed to the atmospheric component at each coupling time step (referred to as FILT). Snapshots of SST illustrate that the frontal-scale SST is mostly removed along the Gulf Stream region, with the half-power wavelength of SST removed by the low-pass boxcar filter being ∼900 km, but with the daily maximum absolute SST gradient nearly unchanged between the twin simulations (Fig. S1 in Supplementary Data). Thus, the feedback of SST perturbations, associated with the Gulf Stream fronts, to the overlying atmosphere is effectively suppressed in FILT without front magnitude heavily reduced. The influence of FOA feedback on STMW production can be assessed by comparing CTRL to FILT.

**Figure 1.**
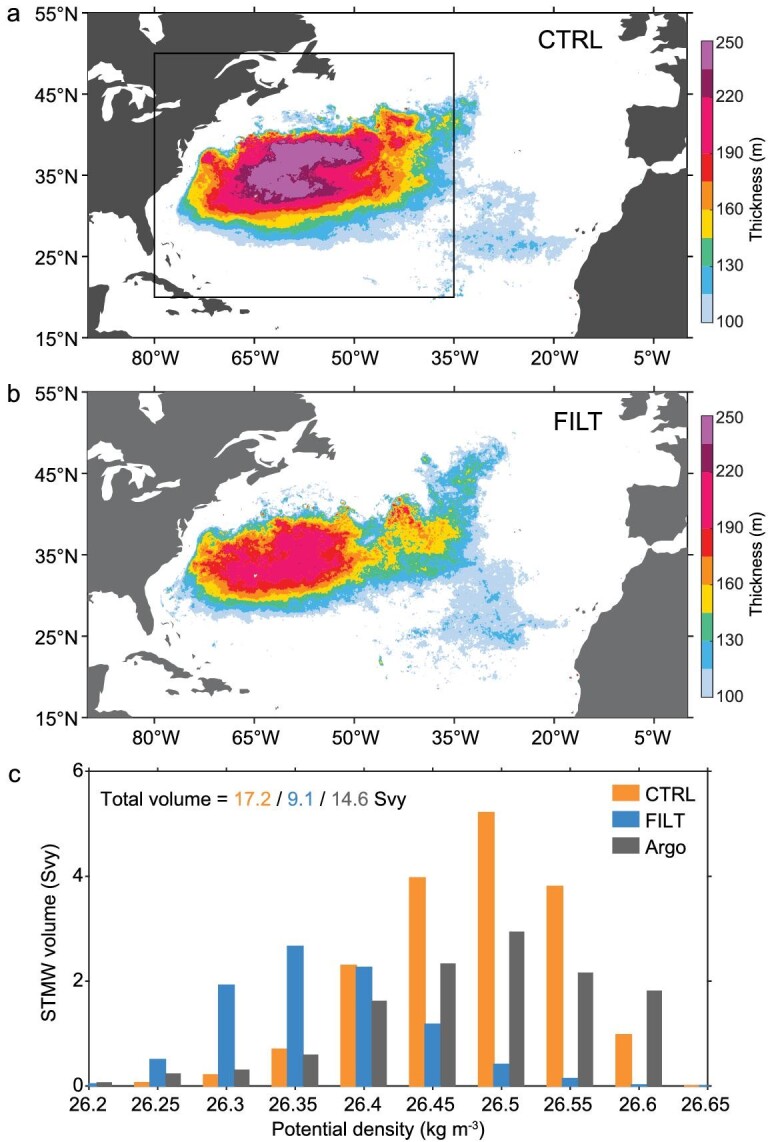
STMW change due to FOA feedback in eddy-resolving global climate simulations. The annual-mean STMW thickness in (a) CTRL and (b) FILT. Box denotes the STMW distribution area (20°–50°N, 80°–35°W). (c) The time-mean volume distribution of STMW for each potential density class (kg m^−3^) based on the CTRL, FILT and IPRC Argo product during 2005–2019. The total volume presented in Svy for the entire density range within each data set is shown on top, where 1 Svy ≈ 3.15 × 10^13^ m^3^ equivalent to 1 Sv (10^6^ m^3^ s^−1^) of volume flux for 1 year.

The reliability of the CESM simulations in capturing STMW, characterized by its low potential vorticity (PV) within the pycnocline, was first verified by comparing vertical profile of PV with three observation-based data sets (Fig. S2; see Observational and reanalysis products in Methods, Supplementary Data). Accordingly, STMW is defined by both PV and potential density constraints using the same criteria for both the observations and CESM simulations (see STMW definition in Methods, Supplementary Data). With full FOA feedback, STMW in CTRL is spread out widely throughout the northwestern part of the subtropical gyre, and its thickness—averaged in the distribution area (20°–50°N, 80°–35°W)—reaches 210.5 m (Fig. 1a), consistent with the estimate based on historical ship-based hydrographic data [2]. In sharp contrast, when FOA feedback is suppressed, STMW in FILT is much thinner, with the distribution-area-averaged thickness reaching 141.3 m (Fig. 1b). By integrating STMW thickness, the total volume unexpectedly decreases by ∼47% in the absence of FOA feedback, i.e. 17.2 Svy in CTRL versus 9.1 Svy in FILT. Further investigation into the volume distribution reveals that STMW with full FOA feedback is produced on denser isopycnals than STMW without full FOA feedback, with the 10-year-mean core layer density reaching 26.36 kg m^−3^ in FILT and increasing to 26.47 kg m^−3^ in CTRL (Fig. 1c). The increase in STMW density is largely accounted for by the upper-ocean cooling, because the core layer temperature is colder by 0.46°C in CTRL than in FILT, while the core layer salinity difference is <0.1 psu between the two simulations. Thus, the presence of FOA feedback leads to a substantial increase in STMW production within denser density classes, which is comparable to the Argo-based estimates of the STMW volume (14.6 Svy) and core layer density (26.51 kg m^−3^).

**Figure 2.**
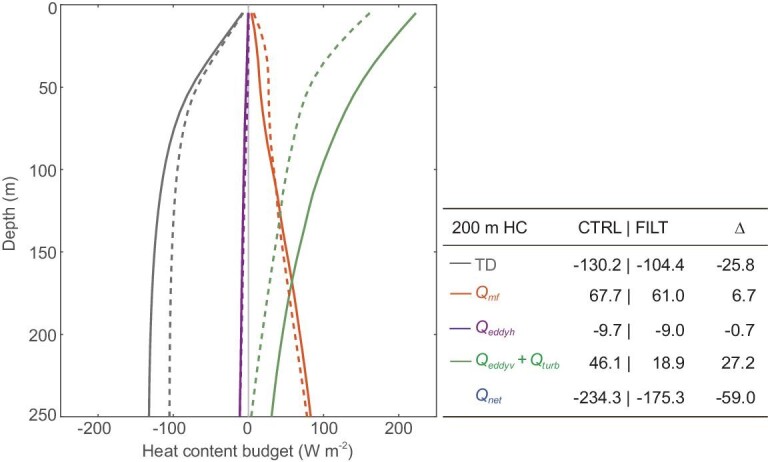
STMW-related heat content change due to FOA feedback. Heat content budget in the upper ocean with TD representing the heat content tendency, *Q_net_* representing the surface net heat flux (positive being downward), *Q_mf_* representing the heat transport convergence by the mean flows, *Q_eddyh_*representing the horizontal eddy heat transport convergence, and *Q_eddyv_*and *Q_turb_* representing the vertical heat transport by eddies and turbulent mixing, respectively. The solid and dashed lines correspond to the budget in CTRL and FILT, respectively. The sub-table shows each budget term integrated in the upper 200 m in CTRL and FILT, with the symbol Δ representing the CTRL minus FILT difference.

The cooling STMW core layer implies a change in the upper-ocean heat content in the presence of FOA feedback. To shed light on the role of surface forcing and internal oceanic processes, we performed the upper-ocean heat content budget analysis based on the twin CESM simulations (see Heat content budget in the upper ocean in Methods, Supplementary Data). The result shows that the presence of FOA feedback leads to a significant decrease in heat content tendency in the upper ocean, with the difference in the upper 200 m layer reaching –25.8 W m^−2^ between CTRL and FILT (Fig. 2). Specifically, the colder tendency with full FOA feedback compared to without full FOA feedback is dominated by the surface heat loss (*Q_net_*, –59.0 W m^−2^), which is partly replenished by vertical eddy heat transport and turbulent vertical mixing (*Q_eddyv_* + *Q_turb_*, 27.2 W m^−2^). The heat transport convergence by the mean flows (*Q_mf_*) and the lateral eddy heat flux (*Q_eddyh_*) are found to have negligible contributions. The increased vertical eddy and turbulent heat transport is a likely consequence of the ageostrophic secondary circulation associated with turbulent thermal wind balance due to the strong surface cooling in the presence of FOA feedback [31]. Thus, the enhanced surface net heat flux with full FOA feedback makes a crucial contribution to decreasing the upper-ocean heat content, resulting in the corresponding colder STMW.

### Enhanced STMW formation traced to surface latent heat loss

**Figure 3.**
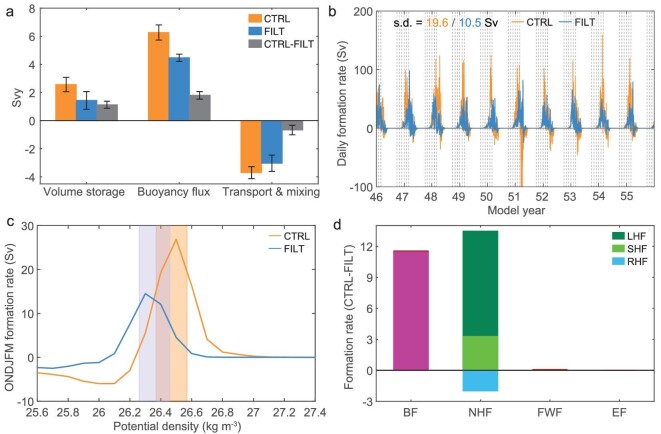
The net annual storage of STMW volume and the STMW formation rate by air-sea fluxes. (a) Annually integrated volume budget for STMW in CTRL, FILT and CTRL minus FILT averaged over the period from October of year 46 to September of year 55, with the terms sequentially representing the annual volume storage, the accumulated water mass formation due to air-sea buoyancy flux, and the accumulated volume consumed by ocean interior diapycnal mixing and volume transport. The error bars denote ±1 standard deviation of the 10 000 inter-realizations based on a bootstrap method. (b) Daily time series of STMW formation rate derived from CTRL and FILT within the respective core layer density ranges as shaded in (c), along with the corresponding daily standard deviation. Vertical dashed lines denote the first day of October-to-March. (c) The wintertime (ONDJFM) mean water mass formation rate for each surface potential density class in CTRL and FILT in the STMW formation region. A positive formation rate corresponds to ocean buoyancy loss, i.e. conversion of lighter water into denser water across a given isopycnal. The yellow and blue shaded areas highlight the STMW density range for CTRL and FILT, respectively. (d) Wintertime-mean STMW formation rates in CTRL minus FILT computed from buoyancy flux (BF), net heat flux (NHF), freshwater flux (FWF), Ekman flux (EF), latent heat flux (LHF), sensible heat flux (SHF) and radiative heat flux (RHF). See Methods for further information.

To probe into the mechanism of increased STMW production due to FOA feedback, we carried out an annually integrated volume budget based on the Walin framework [32] (see Walin formalism in Methods, Supplementary Data). It is evident that the presence of FOA feedback causes an increase (1.1 ± 0.2 Svy) in the annual STMW volume storage, with the average net volume change by year end reaching 2.5 ± 0.5 Svy in CTRL and decreasing to 1.4 ± 0.6 Svy in FILT (Fig. 3a). This increase is statistically significant above the 95% confidence level, according to a bootstrap method (see Bootstrap test in Methods, Supplementary Data). The source of this volume storage difference comes from a significant increase (1.8 ± 0.3 Svy) in the accumulated water mass formation due to air-sea buoyancy flux (6.3 ± 0.5 and 4.5 ± 0.3 Svy in CTRL and FILT, respectively). This externally forced STMW volume production due to air-sea buoyancy flux is partly compensated by the combined effect of both diapycnal mixing and lateral volume transport in the ocean interior. Such volume destruction is modestly higher in CTRL (3.7 ± 0.4 Svy) than in FILT (3.0 ± 0.6 Svy), indicating a potential enhancement of both ocean mixing and advection in the presence of FOA feedback. This is consistent with larger *Q_mf_* and *Q_eddyv_* + *Q_turb_* in CTRL than in FILT (cf. Fig. 2). The greater (8.1 Svy) STMW volume in CTRL than in FILT with respect to the estimated increase rate of annual volume storage (∼1.1 Svy) implies a time span of 7–8 years. It supports the theory that the near-decade-long period of CESM simulation is sufficient for the prominent accumulation of STMW production with full FOA feedback. Thus, it is clear that the increased STMW production in response to FOA feedback can be explained by the corresponding change in water mass formation due to air-sea buoyancy flux. We therefore further probe into the differences between the STMW formation rate (FR) with and without FOA feedback (see Formation rate estimates in Methods, Supplementary Data).

The daily time series of the STMW FR by air-sea buoyancy flux in CTRL exhibits remarkable high-frequency fluctuations, whose amplitude (the daily standard deviation of 19.6 Sv) is almost double that in FILT (10.5 Sv), indicating the much more intense air-sea buoyancy exchange on rather short time scales during STMW formation in the presence of FOA feedback (Fig. 3b). Such high-frequency fluctuations of the STMW FR exhibit positive values during the outcropping season (ONDJFM) with peaks in March due to extensive ocean buoyancy loss, thereby transforming plenty of lighter water masses into STMW. The suppression of FOA feedback leads to approximately only half of lighter water masses being transformed during the outcropping season, with the wintertime-mean FR in the STMW density range reaching 24.5 Sv in CTRL and decreasing to 13.0 Sv in FILT (Fig. 3c). This percentage change in the wintertime FR by air-sea buoyancy flux matches the total STMW volume difference with and without FOA feedback.

A natural question then arises as to what the relative importance of different components of air-sea buoyancy flux is to the increased FR in the presence of FOA feedback. Those components include surface net heat flux, net freshwater flux and Ekman flux driven by wind-induced cross-front advection of density. Clearly, the wintertime-mean STMW formation map by the air-sea buoyancy flux is attributable to that by surface net heat flux, with both being confined to the recirculation gyre region south of the Gulf Stream (Fig. S3a and b in Supplementary Data). The contributions from the freshwater flux and Ekman flux are much less important (Fig. S3c and d in Supplementary Data). Quantitatively, the buoyancy-flux-induced FR response to FOA feedback is almost completely accounted for by its surface net heat flux component (Fig. 3d). In particular, nearly 90% of the increased FR induced by net heat flux is accounted for by the latent heat flux (LHF). Thus, it is the enhanced FR by the wintertime net heat flux, largely by LHF, that leads to the increased STMW volume in the presence of full FOA feedback.

### Mechanism for the LHF-induced increase in STMW formation

**Figure 4.**
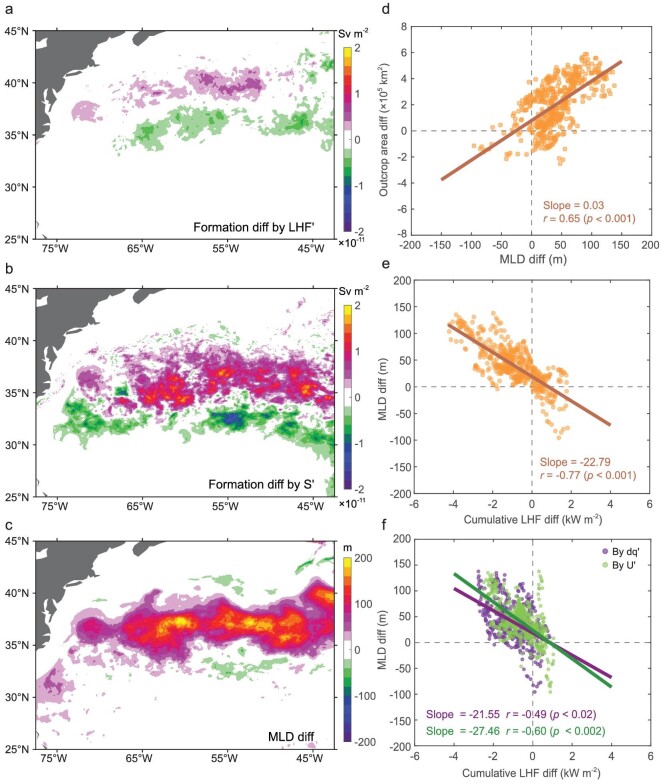
Mechanism for the increased STMW formation rate in the presence of FOA feedback. The wintertime (ONDJFM) mean STMW formation map in CTRL minus FILT difference by (a) LHF difference and by (b) outcropping difference. (c) The late-winter (February–March) mean MLD in CTRL minus FILT difference. (d) Scatter diagram of daily STMW outcrop area difference versus MLD difference induced by FOA feedback in the STMW formation region in February–March from year 46 to year 55. The line denotes a linear fit to these points, of which the slope is shown in the corner of each panel, followed by the correlation coefficient *r* and the corresponding significance *p* value (in parentheses). (e) Same as in (f) but for the daily MLD difference and the cumulative LHF difference accumulated in the 60 days preceding the occurrence of MLD difference. (f) Same as in (e) but for the daily MLD difference and the cumulative LHF difference induced by surface-wind-speed difference and air-sea humidity contrast difference, respectively.

We decomposed the LHF-induced FR difference between CTRL and FILT into the direct contribution from LHF difference and the indirect contribution from STMW outcrop area difference (see Formation rate by latent heat flux in Methods, Supplementary Data). Comparing the wintertime-mean STMW formation difference by these two contributors (Fig. 4a and b), it is found that a considerable increase in surface outcrop area within the formation region dominates the enhanced STMW production in the presence of FOA feedback. In particular, such an outcropping-induced STMW formation difference is primarily driven by the corresponding transformation rate difference across the lighter boundary of STMW, which is further supported by the higher outcrop frequency and enlarged surface outcrop area mainly occurring on the lighter instead of denser boundary of STMW (Fig. S4 in Supplementary Data). The indirect contribution from the increased outcropping (9.98 Sv) almost totally explains the enhanced wintertime STMW FR by LHF, which raises the question as to why the surface outcrop area is enlarged under the context of FOA feedback.

The outcropping of STMW occurs when the corresponding isopycnals intersect the sea surface and the enlargement in outcropping is reminiscent of the mixed layer deepening. Further investigation into the time evolution of the upper-ocean structure in the STMW key region (32°–41°N, 65°–55°W) reveals that surface outcrops in late winter (February–March) and the injection of low PV of STMW into the ocean interior are closely associated with the seasonal deepening of the mixed layer (Fig. S5 in Supplementary Data). As expected, the late-winter mixed layer depth (MLD) in CTRL is significantly deeper (up to 200 m) than that in FILT along the warm flank of the Gulf Stream (Fig. 4c), which coincides with the increased outcropping-induced STMW formation rate in the presence of FOA feedback (Fig. 4b), with the spatial correlation coefficient reaching 0.76. Furthermore, regarding the daily fluctuations, the response of the surface outcrop area to FOA feedback is significantly positively correlated with the MLD response in the STMW formation region (*r* = 0.65, *p* < 0.001; Fig. 4d). This relationship suggests the critical role of deeper mixed layers in more surface outcrops, resulting in higher STMW FR with full FOA feedback than without full FOA feedback.

Given that the higher STMW FR with full FOA feedback lying in an enlarged outcrop is attributable to the deeper mixed layer, a question then arises as to how FOA feedback deepens the mixed layer. Previous studies proposed that the late-winter deepening of the mixed layer on short time scales is primarily controlled by cumulative latent heat release from the ocean [33]. In the STMW formation region, the daily MLD difference between CTRL and FILT is significantly negatively correlated with the cumulative upward LHF difference (*r* = –0.77, *p* < 0.001; Fig. 4e), such that an ∼1 kW m^−2^ increase in the accumulated latent heat release within two months precedes the occurrence of ∼23 m MLD increase in late winter. Hence, the deeper mixed layer in CTRL than FILT is attributable to the cumulative excessive ocean latent heat release driven by FOA feedback. Further decomposition (see LHF reconstruction in Methods, Supplementary Data) shows that the daily MLD difference is related to the cumulative LHF difference caused by changes in both surface wind speed and air-sea humidity contrast, given the comparable negative correlation (Fig. 4f). Specifically, the surface wind speed and the air-sea humidity contrast difference reflect the imprint of frontal-scale SST, with much higher wind speeds and sharper air-sea humidity contrast leading to larger cumulative upward LHF confined to the Gulf Stream front in the context of locally active FOA feedback (Fig. S6 in Supplementary Data). The frontal warmer SST drives sharper air-sea humidity contrast via increased air-sea temperature differences and drives higher wind speeds via the hydrostatic pressure adjustment [25,34] or vertical mixing effect [35,36].

Overall, the considerable transformation of lighter water masses into STMW in the presence of FOA feedback results from a vast increase in its outcrop area accompanied by the deeper mixed layer along the warm flank of the Gulf Stream. The latter is caused by the cumulative excessive ocean latent heat release primarily due to higher surface wind speed and sharper air-sea humidity contrast over the frontal-scale warmer SST.

### Implications for STMW simulation in CGCMs

**Figure 5.**
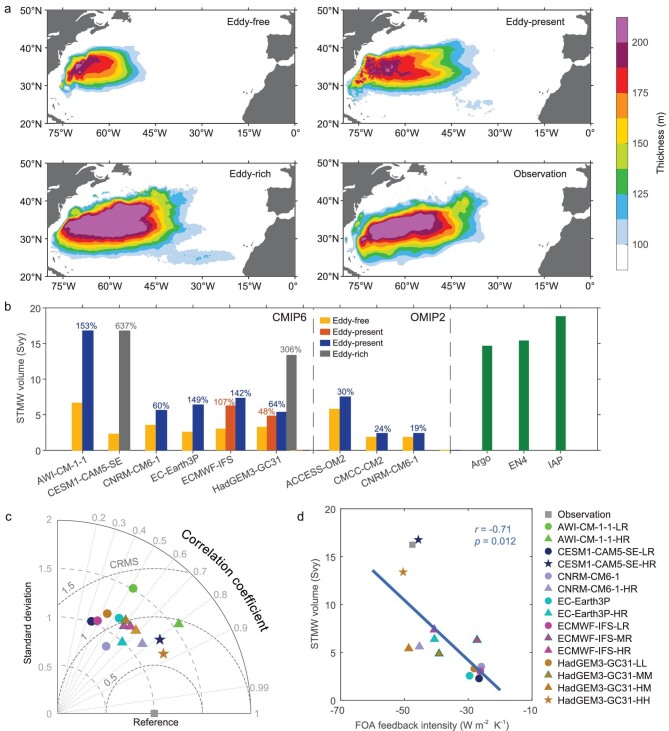
Dependence of STMW representation on model resolution and FOA feedback intensity. (a) Multimodel mean STMW thickness in the CGCMs classified into oceanic eddy-free (≥50 km), eddy-present (∼25 km) and eddy-rich (∼10 km) resolution regimes during 1981–2010, as well as the STMW thickness from the observationally based data sets computed as the averaged thickness based on IPRC Argo (2005–2019), EN4 (1981–2010) and IAP (1981–2010) products. (b) Total volume of STMW in six CGCMs and three OGCMs at different resolutions as well as three observationally based data sets. (c) Taylor diagram for the STMW thickness pattern. Point marked ‘Reference’ refers to the observational pattern in (a). (d) Inter-model relationship between the STMW volume and the wintertime-mean FOA feedback intensity. The linear regression is displayed together with the inter-model correlation coefficient *r* and significance *p* value assessed by Student's *t* test. Dots, triangles and stars signify the eddy-free, eddy-present and eddy-rich configurations of CGCMs, respectively.

STMWs in most CGCMs participating in the Coupled Model Intercomparison Project (CMIP) at standard horizontal resolutions (∼250 km atmosphere and 100 km ocean) are biased towards lower production [28,37]. These coarse resolution regimes, however, are not representative of the vigorous oceanic fronts and eddies as well as their interactions with the atmosphere. Therefore, such a deficiency probably accounts, in part, for the STMW representation biases with insufficient resolution. A piece of evidence to support this assertion can be obtained from the historical simulations of six state-of-the-art CGCMs at different resolutions participating in the High-Resolution Model Intercomparison Project (HighResMIP) of the sixth phase of CMIP (CMIP6) (see STMW simulated by models at different resolutions in Methods, Supplementary Data). According to the oceanic resolution of these CGCMs, historical simulations are classified into three regimes: eddy-free (≥50 km), eddy-present (∼25 km) and eddy-rich (∼10 km). It is clear that the ensemble mean STMW thickness in the eddy-rich simulations agrees significantly better with the observations than those in the eddy-free and eddy-present simulations (Fig. 5a). This result is robust for comparisons among different resolution configurations of each model family (Fig. S7 in Supplementary Data). In particular, the biases of spatial distribution and variation of STMW thickness at coarse resolutions are evidently reduced as the oceanic resolution becomes finer (Fig. 5c).

Critically, the total volumes of STMWs in the eddy-free configurations of six CGCMs are 2.3–6.6 Svy, on average, only one fifth of the observational mean (16.3 Svy) (Fig. 5b). The total volumes greatly increase by 48%–153% in the eddy-present configurations compared to their eddy-free counterparts, and sharply increase by 306% and 673% in the eddy-rich HadGEM3-GC31-HH and CESM1-CAM5-SE-HR, approaching the observational estimates. In contrast, such improvement of STMW production with an increase in oceanic resolution alone substantially degrades to under a third (19%–30%) based on three oceanic general circulation models (OGCMs) participating in the Ocean Model Intercomparison Project phase 2 (OMIP-2). This sharp contrast indicates the key role of the Gulf Stream thermal fronts’ feedback to the overlying atmosphere in controlling STMW production. Indeed, the wintertime-mean FOA feedback intensity in the two eddy-rich CGCMs is also close to the observation (Fig. 5d; see FOA feedback intensity in Methods, Supplementary Data). More importantly, models with higher resolutions tend to generate more realistic and stronger FOA feedback (Fig. S8 in Supplementary Data), which is accompanied by the larger STMW volume. The inter-model correlation is statistically significant (*r* = –0.71, *p* < 0.02), indicating that modeling more realistic FOA feedback is crucial to alleviating the common bias of a too-small STMW volume in CGCMs at standard CMIP resolutions, and lending further support to our CESM experimental findings.

## CONCLUSION AND DISCUSSION

Collectively, our study demonstrates that the feedback of sharp surface thermal fronts, shaped by the Gulf Stream, to the overlying atmosphere (i.e. the local FOA feedback) is essential for STMW formation as it transforms plenty of lighter water masses into STMW through the cumulative extensive latent heat loss and the resultant increased surface outcropping. The enhanced surface heat release into the atmosphere is mainly caused by higher surface wind speed and sharper air-sea humidity contrast driven by the Gulf Stream fronts, and leads to the STMW-related upper-ocean cooling, which is partly compensated by the increased vertical eddy and turbulent heat transport. According to the annually integrated volume budget, the dispersion of STMW is also slightly increased in the presence of FOA feedback, maybe due to the increased lateral transportation by the strengthened Gulf Stream extension current [38]. The mesoscale eddy activity is also relatively weaker with full FOA feedback than without full FOA feedback, because of the enhanced damping of eddy potential energy [38]. This may lead to weaker PV erosion [39] and in turn helps sustain STMW formation.

Recent studies have pointed out changes in large-scale atmospheric circulation under different SST resolutions [40,41], which could further modulate the oceanic fronts associated with strong western boundary currents according to classic wind-driven circulation theory. In fact, the Gulf Stream position does not exhibit significant change in the twin simulations, and the intensity of the large-scale westerly jet is weakened in CTRL compared to FILT (Fig. S9 in Supplementary Data). The latter is likely attributed to the Rossby wave trains emanating from the North Pacific. Such reduced westerly wind, however, does not favor STMW formation. Therefore, the increased STMW production in CTRL is unambiguously caused by local FOA feedback rather than large-scale atmospheric circulation.

We also demonstrate that the eddy-present and eddy-rich CGCMs reproduce more realistic spatial distributions and total volumes of STMWs compared to their low-resolution eddy-free counterparts and OGCMs, due to stronger FOA feedback intensity within the STMW formation region as the model resolution becomes finer. Resolving FOA feedback, therefore, is of paramount importance in reducing the severe underestimation of STMW in most models participating in CMIPs, and would improve representation of STMW’s climatic and biogeochemical impacts. This could be achieved by a coordinated increase in oceanic and atmospheric resolutions or by parameterization of SST front-driven winds in coarse-resolution models [42,43]. The dependence of STMW representation on model resolution also calls for careful evaluation of the projected changes in STMW’s capability for heat storage and carbon sequestration in a warming climate.

## METHODS

Detailed descriptions of methods are available in the Supplementary Data.

## Supplementary Material

nwad133_Supplemental_File

## Data Availability

Data used in this study can be downloaded from IPRC Argo (http://apdrc.soest.hawaii.edu/las/v6/dataset?catitem=208), EN4 v4.2.1 (https://www.metoffice.gov.uk/hadobs/en4/download-en4-2-1.html), IAP (http://159.226.119.60/cheng/), ERA5 (https://cds.climate.copernicus.eu/#!/search?text=ERA5&type=dataset), CMIP6 HighResMIP and OMIP-2 (https://esgf-node.llnl.gov/search/cmip6/). CESM developed by the National Center for Atmospheric Research is freely available as open-source code (https://www.cesm.ucar.edu/models/cesm1.2/). The Matlab is used for plotting.
