# Supplementary material for: North Atlantic subtropical mode water formation controlled by Gulf Stream fronts
Source: Natl Sci Rev. 2023 May 8;10(9):nwad133. doi: 10.1093/nsr/nwad133 (PMC10411678; doi:10.1093/nsr/nwad133)
Supplement: nwad133_Supplemental_File [file nwad133_supplemental_file.pdf]

Supplementary Data for

North Atlantic subtropical mode water formation controlled by Gulf Stream fronts

Bolan Gan[†*], Jingjie Yu[†], Lixin Wu, Gokhan Danabasoglu, R. Justin Small, Allison H. Baker, Fan Jia, Zhao Jing, Xiaohui Ma, Haiyuan Yang, and Zhaohui Chen

[†]These authors contributed equally to this work as co-first authors.
*Corresponding author. Email: gbl0203@ouc.edu.cn

This PDF file includes:

Methods
Figs. S1 to S10
Table S1 to S2
References for Supplementary Data

Methods

Model configuration and experimental design

A set of twin eddy-resolving coupled global climate simulations used in this study was performed at the National Center for Atmospheric Research (NCAR) with a version of the Community Earth System Model (CESM). The model configuration includes the Community Atmosphere Model version 5 (CAM5) with the Spectral Element dynamical core, the Parallel Ocean Program version 2 (POP2), as well as the Community Ice Code version 4 and the Community Land Model version 4. The nominal horizontal resolutions of CAM5 and POP2 are 0.25° and 0.1° (decreasing from 11 km at the equator to 2.5 km at high latitudes), respectively. The control simulation (CTRL) was integrated for 100 years after 14 years of spin-up under "present-day" (year 2000) radiative forcing. The twin filter simulation (FILT) was branched off from 1 March of year 45 of CTRL after spin-up and was integrated for 11 years. Its configuration was the same as CTRL except that a 1,000 km × 1,000 km low-pass boxcar filter was applied to the simulated sea surface temperature (SST) in POP2 before being passed to CAM5 at each coupling time step. This procedure removes the imprints of SST perturbations associated with oceanic fronts and eddies on the atmosphere, which in turn forces the ocean by air-sea surface fluxes without the meso- and frontal-scale SST signatures. Given that the only difference between the twin simulations is whether the atmosphere 'sees' the frontal-scale SST, we compared the results from CTRL and FILT during the 10-year period from year 46 to year 55 to investigate the influence of FOA feedback.

Observational and reanalysis products

Three observationally-based datasets used for STMW calculation are the International Pacific Research Center (IPRC) gridded Argo product spanning from 2005 to 2019, the EN4 version 4.2.1 produced by Met Office Hadley Centre with G10 objective analyses [1], and the Institute of

Atmospheric Physics (IAP) product [2]. Oceanic variables, including temperature and salinity, from all three datasets are objectively-analyzed monthly in situ observations with a horizontal resolution of 1°×1° and vertically interpolated into 10-m intervals using the Akima's scheme [3]. Considering the bias due to sparseness of in situ ocean observations before the 1970s [4], here we calculated STMW in EN4 and IAP for a climatological 30-year period from 1981 to 2010.

To evaluate the FOA feedback intensity, we used the monthly SST and air-sea net heat flux derived from the fifth-generation reanalysis product (ERA5) [5] produced by the European Centre for Medium-Range Weather Forecasts (ECMWF). It has been demonstrated that the high-resolution (horizontal grid spacing of 0.25°) ECMWF ERA5 reanalysis enables the representation of oceanic frontal-scale signals along with oceanic fronts' and eddies' imprints on the overlying atmosphere [6]. The period of 1981–2010 was used for computing the climatological mean state of the FOA feedback intensity around the STMW formation region.

STMW definition

The definition of STMW used in this study aims to identify the pycnostad with a low potential vorticity (PV) constraint in contrast to the strong stratification in the pycnocline. Here, PV $= -\frac{f}{\rho_0}\frac{\partial \rho}{\partial z}$, where f is the Coriolis parameter, ρ_0 is a reference density (1024 kg m^{-3}), ρ is the potential density of seawater, and z is the vertical coordinate, positive upwards. The STMW computed from the Argo and EN4 products and CESM twin simulations is defined as a pycnostad between 26.2–26.6 kg m^{-3} with a PV of $< 1\times10^{-10}$ m^{-1}s^{-1} and a thickness of > 100 m. For the IAP product the potential density constraint of 26.2–26.8 kg m^{-3} was used to encompass the low-PV water mass (Fig. S2).

We depicted the potential density and other properties of STMW by tracking its core layer, which is defined as a local vertical minimum PV within the STMW. The core layer preserves the

winter mixed layer conditions and is regarded as the best record of water mass properties at the time of formation. Following ref. 1, the core layer properties are derived by volume-weighted average of the monthly values in the STMW distribution region during May-to-December when the STMW properties are not destructed apparently by outcropping.

Heat content budget in the upper ocean

The heat content budget for the upper ocean can be derived as:

$$\underbrace{\left\langle \int_{-h}^{0} \rho_0 C_P \frac{\partial \overline{T}}{\partial t} dz \right\rangle}_{TD} = \underbrace{-\left\langle \int_{-h}^{0} \rho_0 C_P \nabla \cdot \left(\overline{\mathbf{u}}\overline{T} \right) dz \right\rangle}_{Q_{mf}} \underbrace{-\left\langle \int_{-h}^{0} \rho_0 C_P \nabla_h \cdot \left(\overline{\mathbf{u}_h{}'T'} \right) dz \right\rangle}_{Q_{eddy_h}} + \underbrace{\left\langle \rho_0 C_P \overline{w'T'} \right\rangle \Big|_{z=-h}}_{Q_{eddy_v}} \quad (1)$$
$$+ \left\langle Q_{turb}\big|_{z=-h} \right\rangle + \left\langle Q_{net} \right\rangle + \left\langle \int_{-h}^{0} \rho_0 C_P \kappa_h \nabla_h^2 \overline{T} dz - \int_{-h}^{0} \rho_0 C_P \kappa_4 \nabla_h^4 \overline{T} dz \right\rangle,$$

where h is the depth for vertical integration, T is the potential temperature, $\mathbf{u} = (\mathbf{u}_h, w)$ is the three-dimensional velocity vector, with $\mathbf{u}_h = (u, v)$ being its horizontal component and w being its vertical component, $\nabla = \left(\partial / \partial x, \partial / \partial y, \partial / \partial z \right)$, $\nabla_h = \left(\partial / \partial x, \partial / \partial y \right)$, κ_h is the horizontal diffusion coefficient, κ_4 is the horizontal biharmonic diffusion coefficient, ρ_0 and C_P are the ocean reference density and heat capacity, respectively. TD represents the heat content tendency, Q_{mf} represents the heat transport convergence by the mean flows, Q_{eddyh} represents the horizontal eddy heat transport convergence, Q_{eddyv} represents the vertical eddy heat transport, Q_{turb} represents the parameterized microscale turbulent mixing, and Q_{net} represents the surface net heat flux (positive into the upper ocean). The heat content budget is conducted for the upper 300 m with the corresponding variables being interpolated onto a regular 10-m vertical grid. Given the negligible effect of horizontal turbulent mixing on the upper ocean heat content [7,8], the last term is not considered in the budget analysis. The overbar denotes the monthly mean values, which are available in the model outputs. The prime denotes the deviation from the monthly mean value,

which can be derived by subtracting the monthly mean values from the model's diagnostic outputs. <…> denotes a spatial average over the STMW formation region plus a time average in the winter season (ONDJFM) from year 46 to year 55.

Water mass formation framework

Walin formalism. We identified and quantified key mechanisms for the difference in STMW production with and without FOA feedback by means of water mass formation framework of Walin [9]. Before this analysis, we verified that the STMW as well as its seasonal cycle can be well defined by the core layer density (σ_{CTRL} = 26.47 kg m^{-3} and σ_{FILT} = 26.36 kg m^{-3}) along with the density windows of $\Delta\sigma = \pm$ 0.1 kg m^{-3}, even without additional constraints on PV or other properties (Fig. S10). This satisfies the prerequisite for exploring mechanisms underlying STMW formation in the Walin framework, which focuses on water masses within a particular density class with no restrictions on stratification [10].

The annually integrated volume budget [11] can be written as:

$$\int_{t_0}^{t_1} \frac{\partial V}{\partial t} dt = \int_{t_0}^{t_1} FR dt + \int_{t_0}^{t_1} D dt - \int_{t_0}^{t_1} M dt \,, \qquad (2)$$

where V is the volume of STMW bounded by the aforementioned isopycnal surfaces $\sigma \pm \Delta\sigma$ and t is time. Term on the left-hand side represents the annual volume storage from the beginning (t_0 = 1 October) to the end (t_1 = 30 September) of a year, when the STMW outcropping starts on 1 October. Terms on the right-hand side correspond sequentially to the accumulated water mass formation due to air-sea buoyancy flux, FR (see next subsection); accumulated diapycnal volume flux due to interior mixing, D; and accumulated volume transport across a control section, M, throughout a year. Here the annual volume storage is approximately computed as the monthly STMW volume in September referenced to the STMW volume in the previous October. The contributions from ocean diapycnal mixing and volume transport are diagnosed as the difference

between the annual volume storage and the accumulated daily formation rate, because the terms comprising daily *D* and *M* were not archived for CESM simulations analyzed here. The annual volume budget is then averaged over the period from October of year 46 to September of year 55.

Formation rate estimates. The total air-sea buoyancy flux *B* (positive being downward) is calculated as the sum of the surface net heat flux (NHF), net freshwater flux (FWF), and Ekman flux (EF) driven by wind-induced cross-front advection of density:

$$B = B_{NHF} + B_{FWF} + B_{EF}$$
$$= \alpha g Q_{net}(\rho_0 C_p)^{-1} - g\beta S(E - P - R) - (\rho_0 f)^{-1}(\vec{\tau} \times \vec{k}) \cdot \nabla b, \qquad (3)$$

where *Qnet* is the surface net heat flux as the sum of sensible heat flux, latent heat flux, and radiative heat flux, *S* is surface salinity, *E* is evaporation, *P* is precipitation, *R* is runoff, $\vec{\tau}$ is wind stress vector, $\vec{k}$ is the unit upward vector, b is buoyancy ($b = -g\Delta\rho/\rho_0$), g is gravitational acceleration, C_p , α, β are the heat capacity, thermal expansion, and haline contraction coefficient of seawater, respectively. Positive buoyancy flux implies ocean buoyancy gain (i.e., a decrease in surface density).

The water mass transformation rate *TR* by the air-sea fluxes is calculated by integrating *B* over the outcrop window bounded by isopycnal surfaces $\sigma \pm \Delta\sigma/2$. The discretized expression is given by:

$$TR(\sigma, t) = \frac{\rho_0}{g\Delta\sigma}\left(\iint_{\sigma_0}^{\sigma+\frac{\Delta\sigma}{2}} B ds - \iint_{\sigma_0}^{\sigma-\frac{\Delta\sigma}{2}} B ds\right) = \frac{\rho_0}{g\Delta\sigma}\iint_{\sigma-\frac{\Delta\sigma}{2}}^{\sigma+\frac{\Delta\sigma}{2}} B ds, \qquad (4)$$

where *ds* is an area element at the sea surface. The instantaneous map of transformation can be expressed by $\rho_0(g\Delta\sigma)^{-1}B\delta(\sigma,\Delta\sigma)$ with $\delta(\sigma,\Delta\sigma)$ being the top-hat function of σ which is zero except within the interval of $\Delta\sigma/2$, where it has unit value [12]. Positive transformation corresponds to volume flux towards increasing density. The convergence of *TR* drives net

subduction across the surface into the ocean interior and yields the rate at which a given density class ($\sigma_1 < \sigma < \sigma_2$) is created or destroyed via air-sea buoyancy flux (i.e., the formation rate *FR*):

$$FR(\sigma, t) = TR(\sigma_1, t) - TR(\sigma_2, t). \tag{5}$$

To estimate the *FR* of STMW, we used the daily *B* and surface density field for the Walin analysis. *B* was integrated over the individual daily outcrop window with the width $\Delta\sigma/2 = 0.05$ kg m^{-3} for STMW. *FR* was then calculated as the difference of the transformation rate bounded by the aforementioned core layer density surfaces 26.47 ± 0.1 and 26.36 ± 0.1 kg m^{-3} that well depict the simulated STMW in CTRL and FILT, respectively, and integrated in the STMW formation regions (32°–41°N for CTRL and 30°–38°N for FILT, 75°–45°W for both).

Formation rate by latent heat flux (LHF). The LHF-induced *FR* difference between CTRL and FILT can be approximated as:

$$\begin{aligned} \Delta FR^{LHF} &= FR^{LHF}(\sigma_{CTRL} \pm \Delta\sigma, t) - FR^{LHF}(\sigma_{FILT} \pm \Delta\sigma, t) \\ &= \frac{\alpha}{C_p \Delta\sigma} \iint_C LHF_C \, ds - \frac{\alpha}{C_p \Delta\sigma} \iint_F LHF_F \, ds \\ &= \frac{\alpha}{C_p \Delta\sigma} \iint_C LHF' \, ds + \frac{\alpha}{C_p \Delta\sigma} \iint_{s\prime} LHF_F \, ds, \end{aligned} \tag{6}$$

where $LHF' = LHF_C - LHF_F$, $s' = outcrop_C - outcrop_F$, the subscript *C* and *F* denote CTRL and FILT, respectively. The terms on the right-hand side of equation (6) represent the contributions from differences in LHF and surface outcrops between CTRL and FILT, respectively. The surface outcrops difference depends on differences in outcrop areas within the window centered at the lower and upper bounds of STMW density between CTRL and FILT.

LHF reconstruction

We reconstructed LHF from surface wind speed (*U*) and air-sea humidity contrast (*dq*) using the following bulk formulae [13]:

$$LHF = \rho_a L_e C_e U dq = \rho_a L_e C_e U\big(q_a - q_s(T_s)\big), \quad (7)$$

where ρ_a is the atmospheric surface density, L_e is the latent heat of vaporization as a function of surface temperature (T_s), expressed as $L_e = (2.501 - 0.00237 \times T_s) \times 10^6$, C_e is the stability dependent turbulent exchange coefficient for latent heat, q_s is the saturation specific humidity at T_s, and q_a is the atmospheric specific humidity at the lowest level. Here, the coefficients are all computed from the Coupled Ocean-Atmosphere Response Experiment version 3.0 (COARE 3.0) bulk algorithm. Negative LHF signifies latent heat release from the ocean to the atmosphere. The reliability of the bulk algorithm in reconstructing LHF has been verified by comparing with the model output of LHF in the twin simulations.

Based on the LHF reconstruction, the LHF difference between CTRL and FILT can be approximated as:

$$LHF' = \rho_a L_e C_e (U' dq_F + U_F dq' + U' dq'), \quad (8)$$

where $U' = U_C - U_F$, and $dq' = dq_C - dq_F$. Because the differences in ρ_a, L_e, and C_e between CTRL and FILT have much smaller magnitudes compared to their respective values [less than $O(10^{-1})$], here we focus on the contributions of surface wind difference (U') and air-sea humidity contrast difference (dq') to the LHF difference with the relevant coefficients regarded as constant.

STMW simulated by models at different resolutions

The dependence of STMW representation on model resolution is assessed using the "hist-1950" experiments from six CGCMs participating in HighResMIP with three-dimensional temperature and salinity outputs available. Each participating model conducted historical simulations at different horizontal resolutions which are classified into three regimes according to the resolution of their ocean components: eddy-free (≥50 km), eddy-present (~25 km), and eddy-rich (~10 km). In general, models with finer oceanic resolution are typically accompanied by higher atmospheric

resolution. The 15 multi-resolution simulations used in this study include 6 eddy-free, 7 eddy-present, and 2 eddy-rich simulations (Table S1). Given different density stratifications in each model, we adjusted the PV threshold and the potential density constraint to define STMWs in individual models appropriately.

The improvement of STMW simulation with increasing ocean resolution alone based on oceanic general circulation models (OGCMs) participating in Ocean Model Intercomparison Project phase 2 (OMIP-2) were used to make comparison with that in CGCMs in order to understand the role of FOA feedback in STMW production. The three OGCMs used in this study (Table S2) are forced by the Japanese 55-year atmospheric reanalysis (JRA-55) [14] in the absence of its response to oceanic feedback, covering the period from 1958 to 2018. Please refer to ref. [15] for more details of the experimental design.

FOA feedback intensity

To measure the intensity of FOA feedback in CGCMs, we regressed the wintertime (ONDJFM) monthly frontal-scale net heat flux onto the frontal-scale SST:

$$C_{FOA} = Cov[NHF'(t), SST'(t)]/Cov[SST'(t), SST'(t)], \quad (9)$$

where the regression coefficient C_{FOA} denotes the FOA feedback intensity. The prime denotes the frontal-scale anomaly computed as the spatially high-pass filtered field achieved by removing a 5°×5° boxcar running mean. We focused on inter-model differences in the total STMW volume and its relationship with the wintertime mean FOA feedback intensity, area-averaged in the key region (29°–41°N, 75°–38°W) where mode water is produced and FOA feedback is very strong across models. We confirmed that the main results in this study are insensitive to modest changes in either the spatial extent of the region used for area averaging or the box size used for spatial filter, consistent with the fact that C_{FOA} remains relatively stable on spatial wavelengths < 1000

km [16]. The AWI-CM-1-1 was not used in the comparison of FOA feedback intensity because of unavailability of heat flux data.

Bootstrap test

A bootstrap method [17] is used to examine whether the differences of STMW volume budget terms between CESM CTRL and FILT are statistically significant. Each term is resampled randomly to conduct 10,000 realizations of mean value for CTRL and FILT (Fig. 3a). In this resampling process, any annual mean value can be selected more than once. The standard deviation of the 10,000 inter-realizations of mean value for each simulation is then computed. If the mean value difference between CTRL and FILT is greater than the sum of those two standard deviations, then it is statistically significant above the 95% confidence level.

Taylor diagram

We used Taylor diagrams [18] to elucidate the capability of CGCMs at different resolutions in reproducing the observationally-based STMW thickness pattern determined as the averaged pattern of the IPRC Argo (2005–2019), EN4 (1981–2010), and IAP (1981–2010) products. Three statistics are calculated to quantify the pattern similarity within the STMW distribution regions: the spatial correlation coefficient, the spatial standard deviation, and the centered pattern root-mean-square (CRMS) difference that incorporates the evaluation of similarities of spatial structure and variation between model and observations. Both the spatial standard deviation and CRMS are normalized by the observational standard deviation to facilitate direct comparison. Therefore, lying closer to the observations indicates a more realistically simulated STMW in CGCMs.

Percentage change

The percentage change in STMW thickness, volume, and formation rate when FOA feedback is suppressed are calculated as: $(FILT - CTRL)/CTRL \times 100\%$. The percentage changes based on

the different resolutions of each HighResMIP model family are calculated relative to the eddy-free counterpart.

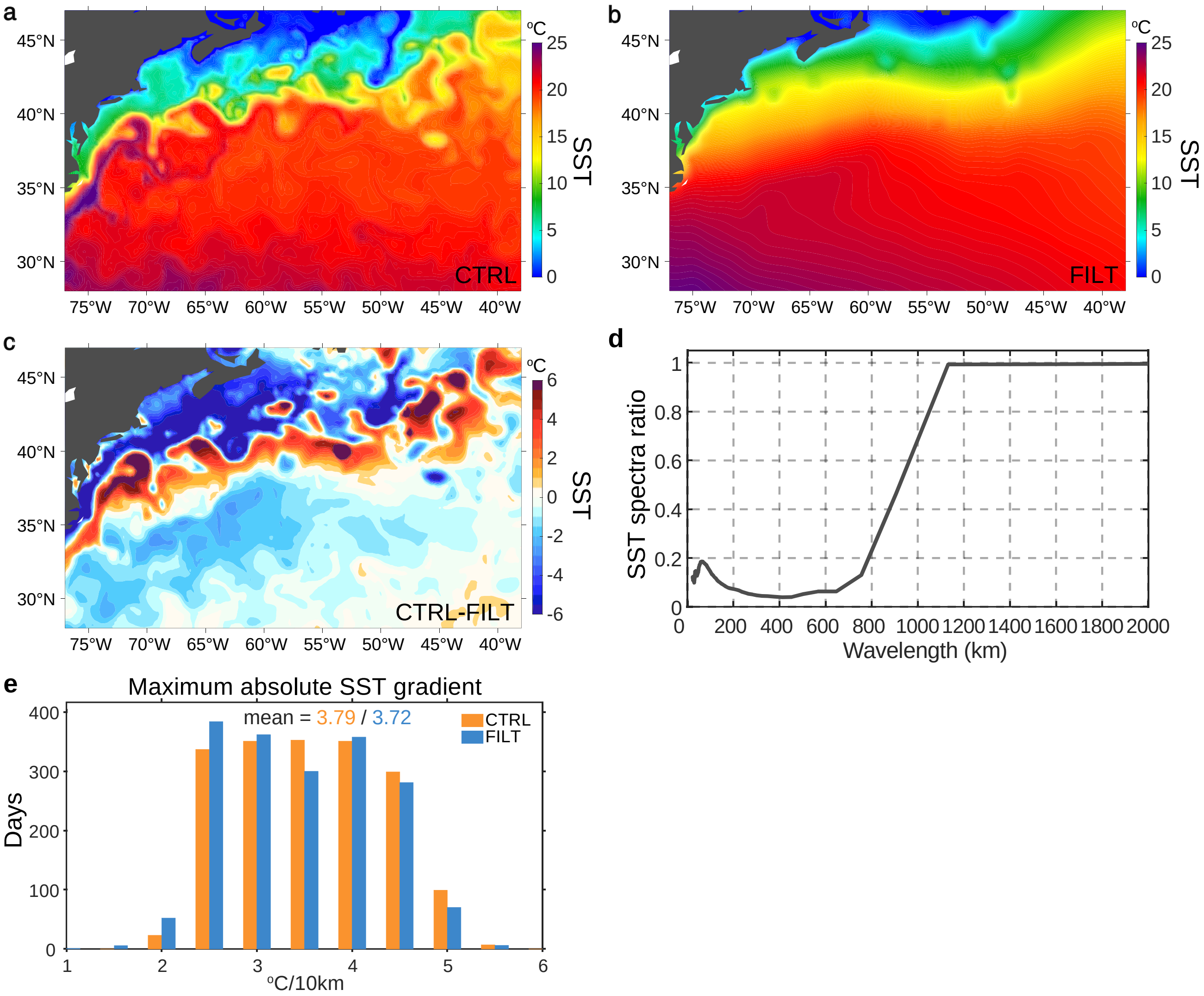

Fig. S1. Comparison of SST in the CESM twin simulations. Snapshots of SST in (a) CTRL, (b) FILT, and (c) CTRL minus FILT difference on February 17 of year 49. **(d)** Wintertime (ONDJFM) mean ratio (FILT/CTRL) of the SST power spectra in the Gulf Stream region (30°–45°N, 75°–40°W). **(e)** Histogram of the wintertime maximum absolute SST gradient that the atmosphere 'sees' in CTRL (yellow bars) and FILT (blue bars) over the Gulf Stream region.

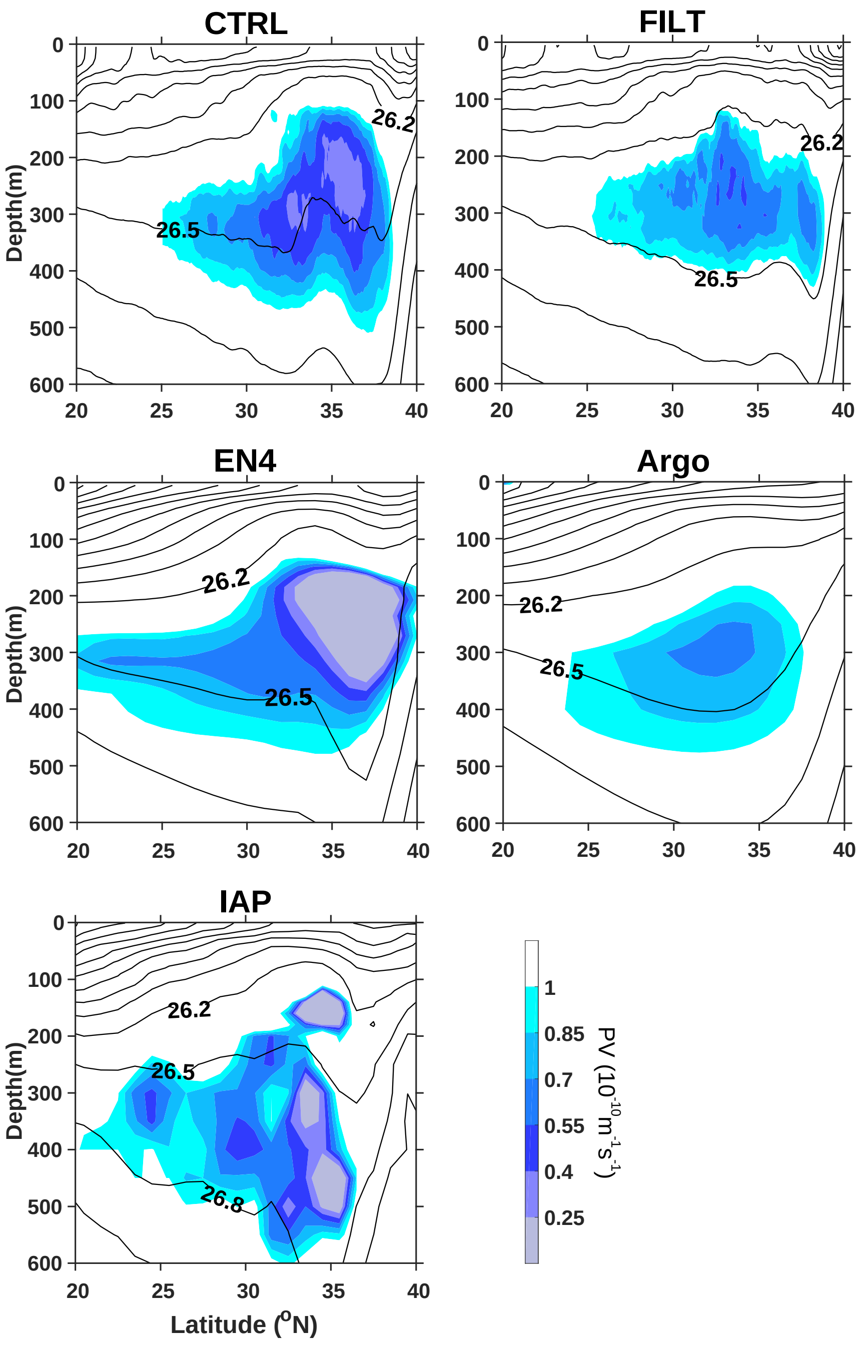

Fig. S2. Meridional sections of low-PV pool in the CESM twin simulations and observations / reanalysis. Typical meridional section (60°W) in June of potential density (black contours in 0.3 kg m^{-3} intervals) and PV (shaded) for STMW from CTRL and FILT, and from the Argo (2005–2019), EN4 (1981–2010), and IAP (1981–2010) products.

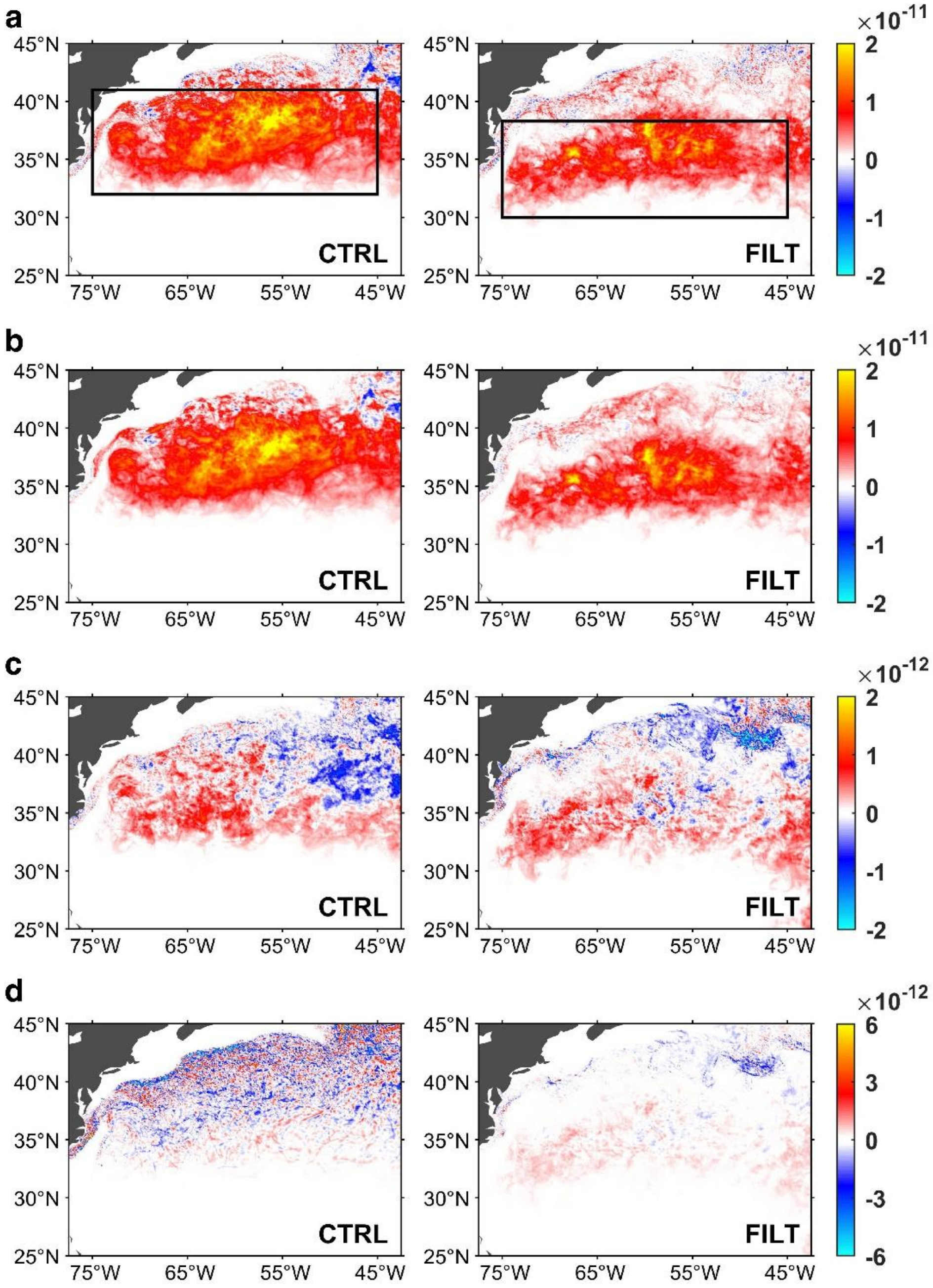

Fig. S3. STMW formation map by air-sea buoyancy flux and its three components in the CESM twin simulations. The wintertime (ONDJFM) mean STMW formation maps by **(a)** buoyancy flux, **(b)** net heat flux, **(c)** freshwater flux, and **(d)** Ekman flux in (left) CTRL and (right) FILT. Black box denotes the main STMW formation region (32°–41°N and 30°–38°N for CTRL and FILT, respectively, and 75°–45°W for both). Note the different color bar scales for different components. Unit is Sv m^{-2}.

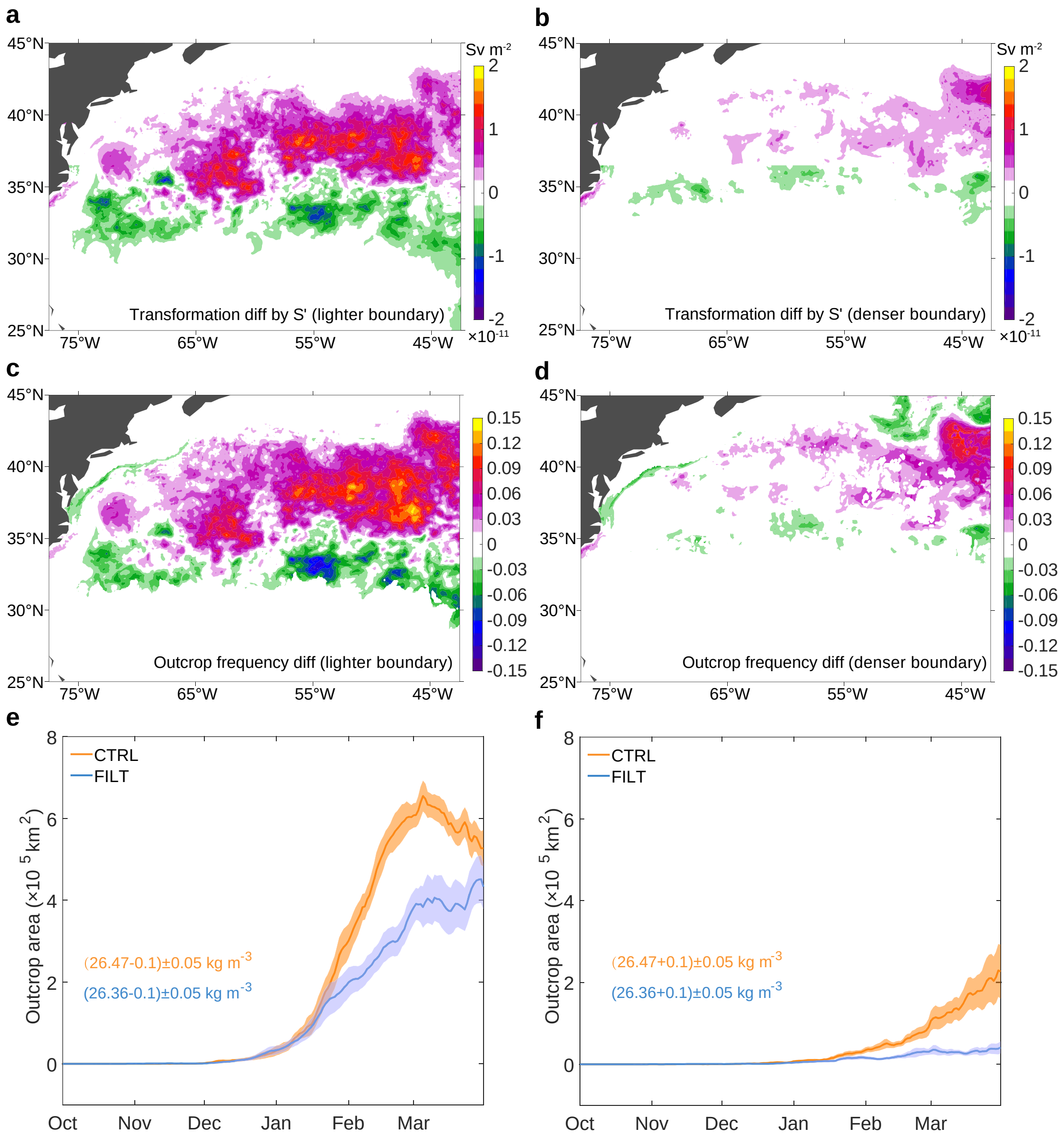

Fig. S4. Comparison of STMW outcropping and corresponding transformation rate between CTRL and FILT. The wintertime (ONDJFM) mean **(a)** STMW transformation map by outcropping difference and **(c)** STMW outcrop frequency at lighter boundary in CTRL minus FILT. **(e)** The corresponding daily climatological outcrop area of STMW bounded by isopycnals σ = (26.47−0.1) ± 0.05 kg m^{-3} for CTRL (yellow curve) and σ = (26.36−0.1) ± 0.05 kg m^{-3} for FILT (blue curve). The shaded areas are the 95% confidence intervals computed from the bootstrap method. **(b), (d), (f)** Same as **(a), (c), (e),** but for the denser boundary of STMW with σ = (26.47+0.1) ± 0.05 kg m^{-3} for CTRL and σ = (26.36+0.1) ± 0.05 kg m^{-3} for FILT.

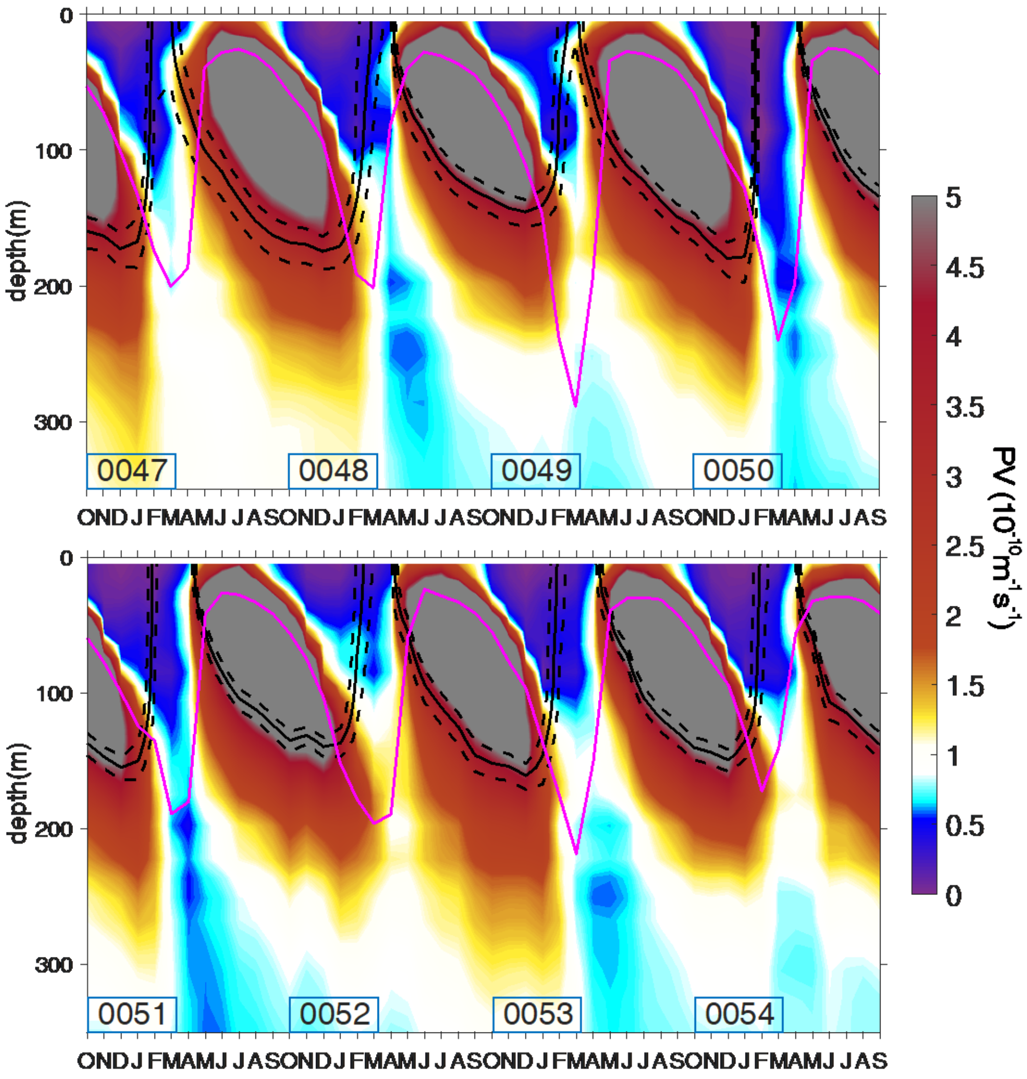

Fig. S5. Time evolution of PV, mixed layer depth, and STMW outcropping. The monthly time series of PV (shaded), along with the base of the mixed layer (m; pink curve) and the depth of the σ = 26.37 kg m^{-3} isopycnal (black curve) with the density windows of ±0.05 kg m^{-3} (black dashed curve), area-averaged in the STMW key region (32°–41°N, 65°–55°W) in CTRL. Note that the results are insensitive to the choice of region.

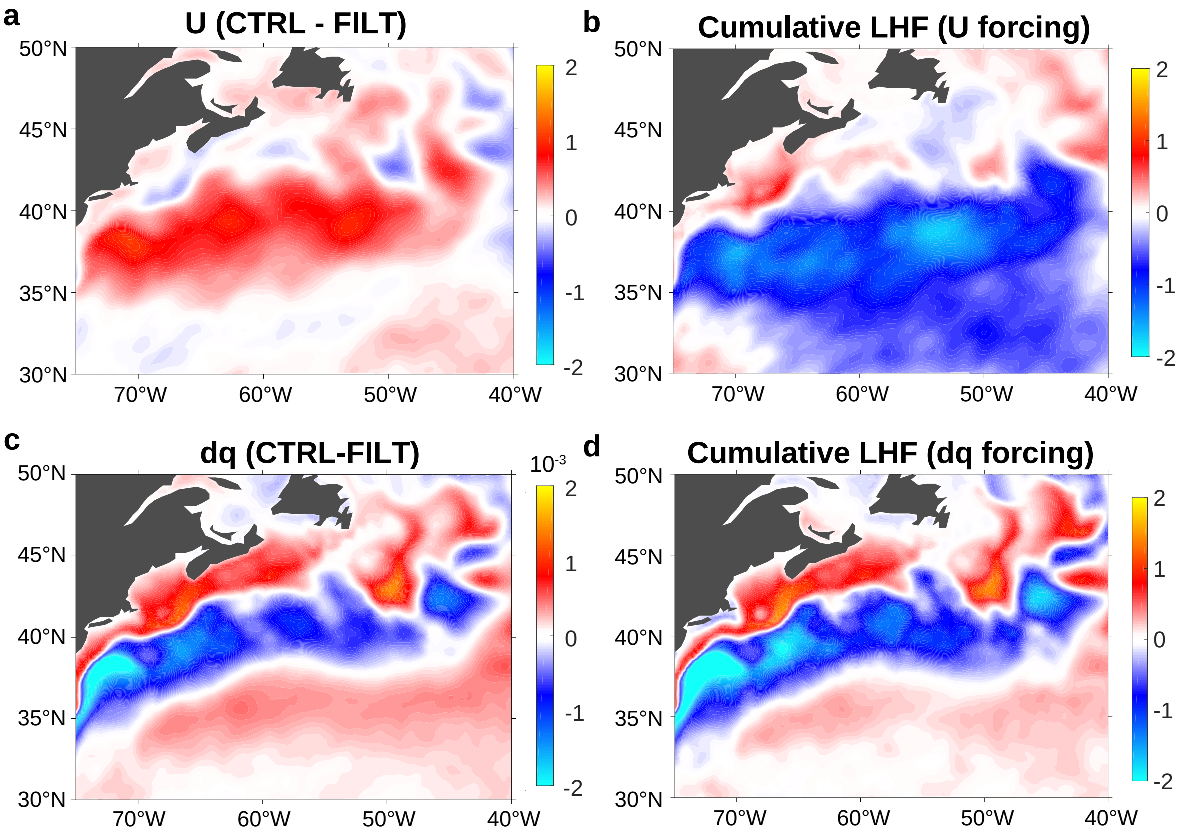

Fig. S6. Surface wind and air-sea humidity contrast response to FOA feedback. The wintertime (ONDJFM) mean difference of **(a)** surface wind speed (m s^{-1}) and **(c)** air-sea humidity contrast (kg/kg) in CTRL minus FILT. The cumulative LHF difference (kW m^{-2}; positive being downward) induced by **(b)** surface wind speed difference and **(d)** air-sea humidity contrast difference, accumulated from 60 days preceding the occurrence of MLD difference in late winter (February–March).

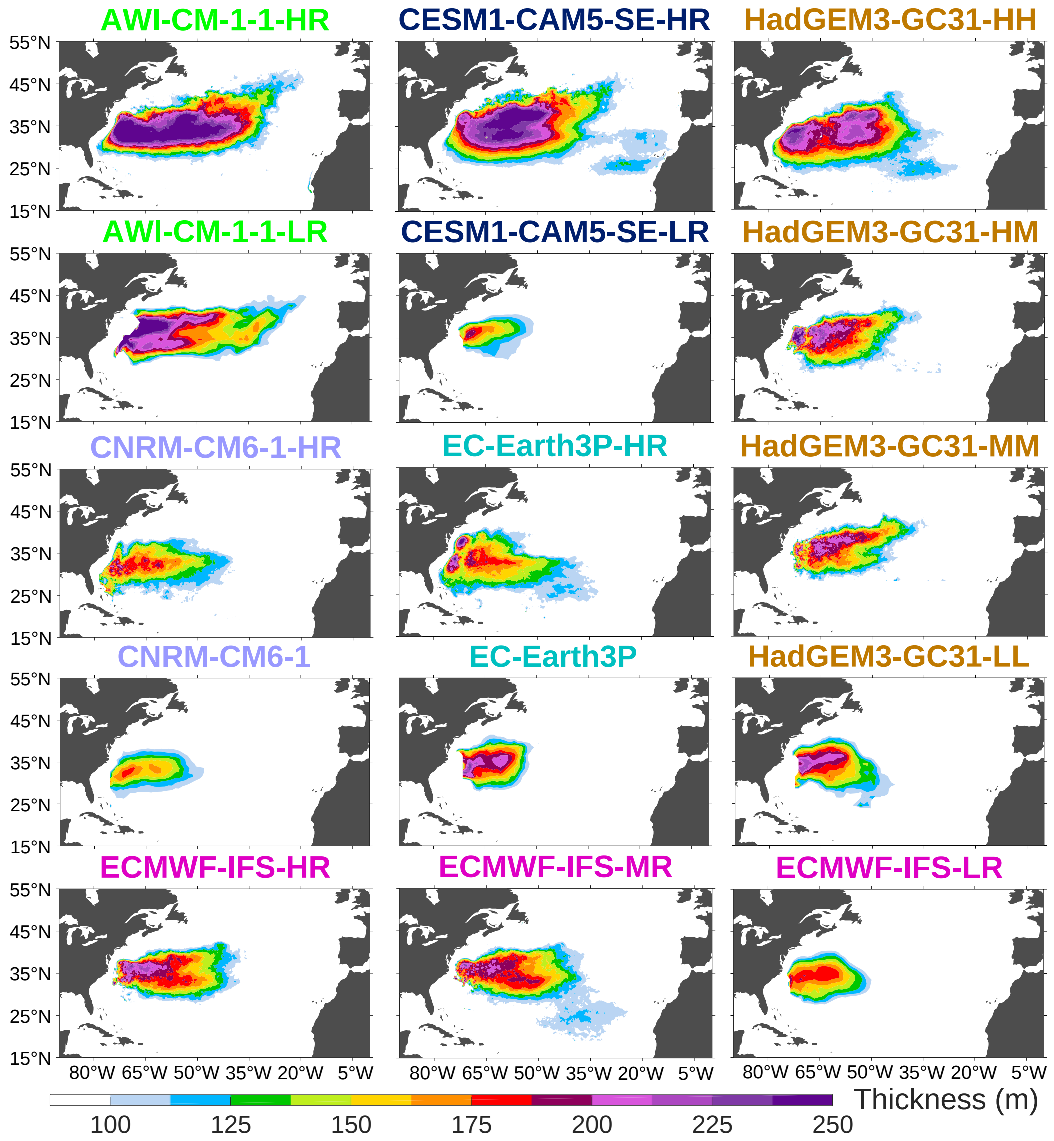

Fig. S7. STMW thickness simulated in CGCMs at different resolutions. The climatological thickness patterns of STMW derived from six HighResMIP models with different horizontal resolutions during 1981–2010. See Table S1 for further information.

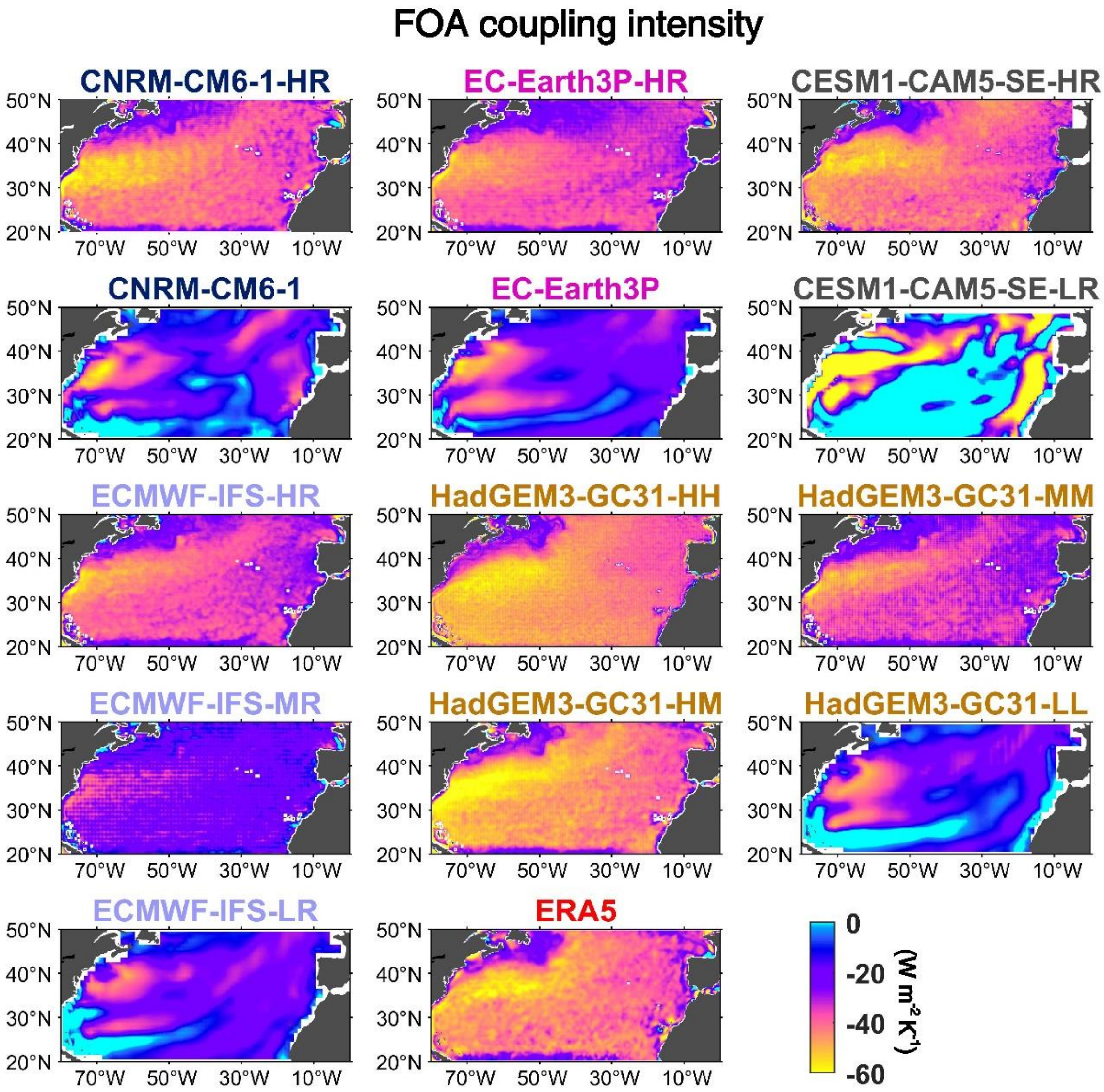

Fig. S8. The FOA coupling intensity in CMIP6 HighResMIP experiments and ERA5 product. The distribution of FOA coupling over the North Atlantic region in five CMIP6 models participating in HighResMIP and ERA5 product during 1981–2010.

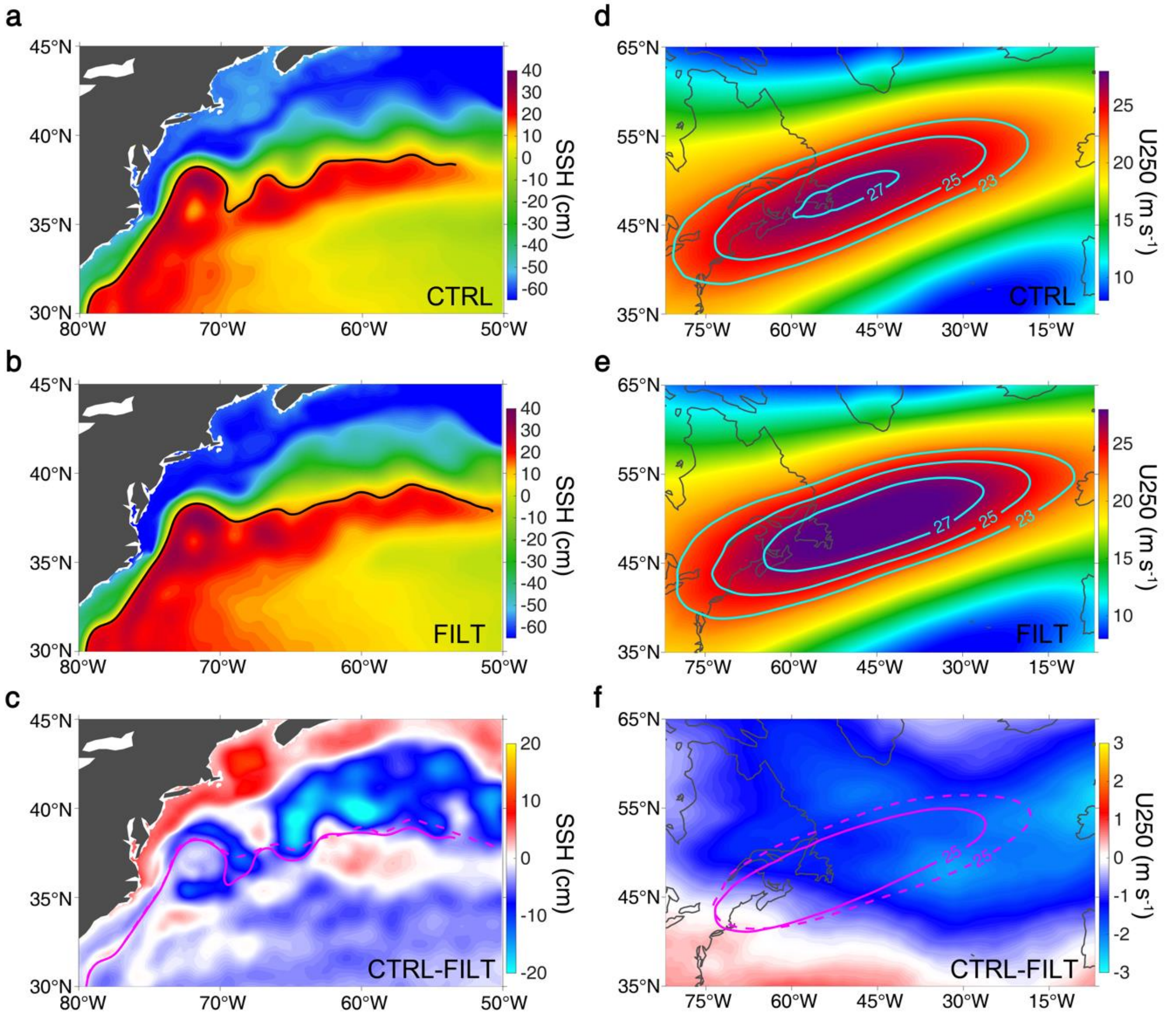

Fig. S9. Gulf Stream position and large-scale atmospheric circulation in the CESM twin simulations. Climatological sea surface height (SSH) simulated in **(a)** CTRL, **(b)** FILT and **(c)** CTRL minus FILT. Thick lines denote the Gulf Stream path based on the 15-cm SSH contours. **(d)–(f)** Same as **(a)–(c)**, but for the climatological zonal wind speed at 250 hPa, with the 25 m s^{-1} contours denoting the upper-tropospheric westerly jet.

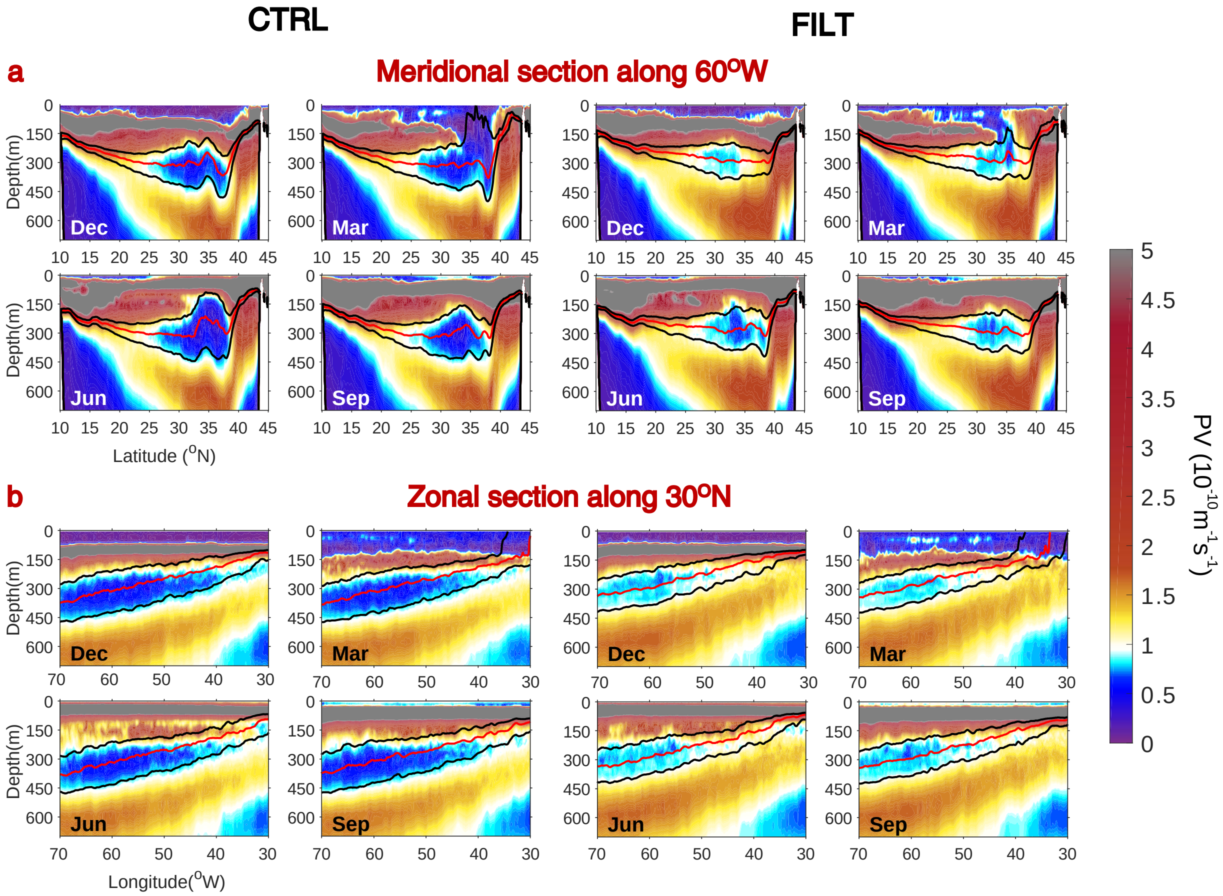

Fig. S10. Comparison of meridional and zonal PV sections between CTRL and FILT for characteristic months of a typical seasonal cycle. Time-mean **(a)** meridional PV section along 60°W for December, March, June, and September in (first two columns) CTRL and (last two columns) FILT. The red and black contours denote the STMW core layer density as σ_{CTRL} = 26.47 kg m^{-3} and σ_{FILT} = 26.36 kg m^{-3} with the density windows of ±0.1 kg m^{-3}, respectively. **(b)** Same as **(a)** but for the zonal PV section along 30°N.

Table S1. List of CGCMs from CMIP6 HighResMIP analyzed in this study.

Models	Configuration	Nominal resolution (atm × ocn)	Ocean model regime
AWI-CM (Alfred Wegener Institute Climate Model)	AWI-CM-1-1-HR	100km × 25km	eddy-present
	AWI-CM-1-1-LR	250km × 50km	eddy-free
CESM (Community Earth System Model)	CESM1-CAM5-SE-HR	25km × 10km	eddy-rich
	CESM1-CAM5-SE-LR	100km × 100km	eddy-free
CNRM-CM (Centre National de Recherches Météorologiques – Earth System Model)	CNRM-CM6-1-HR	100km ×25km	eddy-present
	CNRM-CM6-1	250km ×100km	eddy-free
EC-Earth3P (EC-Earth-Consortium)	EC-Earth3P-HR	50km ×25km	eddy-present
	EC-Earth3P	100km ×100km	eddy-free
ECMWF-IFS (European Centre for Medium-Range Weather Forecasts – Integrated Forecasting System Model)	ECMWF-IFS-HR	25km × 25km	eddy-present
	ECMWF-IFS-MR	50km × 25km	eddy-present
	ECMWF-IFS-LR	50km × 100km	eddy-free
HadGEM-GC31 (Hadley Global Environment Model – Global Coupled configuration 3.1)	HadGEM3-GC31-HH	50km ×10km	eddy-rich
	HadGEM3-GC31-HM	50km × 25km	eddy-present
	HadGEM3-GC31-MM	100km × 25km	eddy-present
	HadGEM3-GC31-LL	250km ×100km	eddy-free

Table S2. List of OGCMs participating in OMIP-2 analyzed in this study.

Models	Configuration	Ocean resolution	Ocean model regime
ACCESS-OM2 (Australian Community Climate and Earth System Simulator – ocean-sea-ice model)	ACCESS-OM2-025<br>ACCESS-OM2	25km<br>100km	eddy-present<br>eddy-free
CMCC-CM2 (Centro Euro-Mediterraneo sui Cambiamenti Climatici - Climate Model Version 2)	CMCC-CM2-HR4<br>CMCC-CM2-SR5	25km<br>100km	eddy-present<br>eddy-free
CNRM-CM (Centre National de Recherches Météorologiques – Earth System Model)	CNRM-CM6-1-HR<br>CNRM-CM6-1	25km<br>100km	eddy-present<br>eddy-free

References for Supplementary Data

1. Good SA, Martin MJ and Rayner NA. EN4: Quality controlled ocean temperature and salinity profiles and monthly objective analyses with uncertainty estimates. *J Geophys Res Oceans* 2013; **118**: 6704–16.
2. Cheng LJ, Trenberth KE and Fasullo J *et al.* Improved estimates of ocean heat content from 1960–2015. *Sci Adv* 2017; **3**: e1601545.
3. Akima H. A new method of interpolation and smooth curve fitting based on local procedures. *J Assoc Comput Mach* 1970; **17**: 589–602.
4. Domingues CM, Church JA and White NJ *et al.* Improved estimates of upper-ocean warming and multi-decadal sea-level rise. *Nature* 2008; **453**: 1090–3.
5. Hersbach H, Bell B and Berrisford P *et al.* The ERA5 global reanalysis. *Quart J Roy Meteor Soc* 2020; **146**: 1999–2049.
6. Pezzi LP, de Souza RB and Santini MF *et al.* Oceanic eddy-induced modifications to air-sea heat and CO_2 fluxes in the Brazil-Malvinas Confluence. *Sci Rep* 2021; **11**: 10648.
7. Jing Z, Wang SP and Wu LX *et al.* Maintenance of mid-latitude oceanic fronts by mesoscale eddies. *Sci Adv* 2020; **6**: eaba7880.
8. Shan X, Jing Z and Gan BL *et al.* Surface heat flux induced by mesoscale eddies cools the Kuroshio-Oyashio Extension region. *Geophys Res Lett* 2020; **47**: e2019GL086050.
9. Walin G. On the relation between sea-surface heat flow and thermal circulation in the ocean. *Tellus* 1982; **34**: 187–95.
10. Maze G and Marshall J. Diagnosing the observed seasonal cycle of Atlantic subtropical mode water using potential vorticity and its attendant theorems. *J Phys Oceanogr* 2011; **41**: 1986–99.
11. Carlson CA, Hansell DA and Nelson NB *et al.* Dissolved organic carbon export and subsequent remineralization in the mesopelagic and bathypelagic realms of the North Atlantic basin. *Deep Sea Res Part II* 2010; **57**: 1433–45.
12. Maze G, Forget G and Buckley M *et al.* Using transformation and formation maps to study the role of air-sea heat fluxes in North Atlantic Eighteen Degree Water formation. *J Phys Oceanog*r 2009; **39**: 1818–35.
13. Fairall CW, Bradley EF and Hare JE *et al.* Bulk parameterization of air-sea fluxes: Updates and verification for the COARE algorithm. *J Clim* 2003; **16**: 571–91.

14. Kobayashi S, Ota Y and Harada Y *et al.* The JRA-55 Reanalysis: General specifications and basic characteristics. *J Meteorological Soc Jpn* 2015; **93**: 5–48.
15. Griffies SM, Danabasoglu G and Durack PJ *et al.* OMIP contribution to CMIP6: Experimental and diagnostic protocol for the physical component of the Ocean Model Intercomparison project. *Geosci Model Dev* 2016; **9**: 3231–96.
16. Yang PR, Jing Z and Wu LX. An assessment of representation of oceanic mesoscale eddy-atmosphere interaction in the current generation of general circulation models and reanalyses. *Geophys Res Lett* 2018; **45**: 11856–65.
17. Austin PC and Tu JV. Bootstrap methods for developing predictive models. *Am Sta*t 2004; **58**: 131–7.
18. Taylor KE. Summarizing multiple aspects of model performance in a single diagram. *J Geophys Res* 2001; **106**: 7183–92.
